# A checklist for designing health insurance programmes – a proposed guidelines for Nigerian states

**DOI:** 10.1186/s12961-019-0480-8

**Published:** 2019-08-22

**Authors:** Yewande Kofoworola Ogundeji, Kelechi Ohiri, Azara Agidani

**Affiliations:** Health Strategy and Delivery Foundation, 1980 Wikki Spring street, Maitama, Abuja, Nigeria

**Keywords:** Social health insurance, health financing, strategic purchasing, benefit package, resource pooling, provider payment, checklist, design

## Abstract

**Background:**

There is widespread and growing interest in designing and implementing social health insurance schemes (SHIS) across many low- and middle-income countries as a means to improve financial protection and achieve universal health coverage. SHIS recently gained traction in Nigeria, but evidence regarding optimal design features of SHIS is sparse and there is lack of a simple and standardised checklist that scheme designers, implementers and researchers could use to assess, guide and inform the design of SHIS. This paper seeks to develop a checklist based on concepts as well as theoretical and empirical evidence that can inform and guide scheme designers and implementers on design options to maximise the effectiveness of the scheme.

**Methods:**

We conducted a review of literature exploring the relevant concepts for the development of a framework and checklist to identify the key factors or variables required to inform the design of SHIS. The checklist details critical considerations/questions to address and options for design. The developed checklist was then used to examine conditions for readiness and appropriateness of SHIS design in two states in Nigeria (Kaduna and Niger).

**Results:**

This paper describes the development of a SHIS checklist. The findings also demonstrate that the newly developed checklist, consisting of six design domains, can be used by scheme designers and policy-makers as a simple and effective tool to assess and inform SHIS design features across Nigeria to maximise the chances of the effectiveness of the schemes.

**Conclusion:**

In conclusion, given that the development of SHIS in the Nigerian states is still in its early stages, applying the SHIS design checklist can serve as a first step to ensuring a feasible and sustainable insurance scheme. The introduction of SHIS, if properly designed and implemented, can be a significant first step towards improving the accessibility, equity and efficiency of healthcare in Nigeria.

## Background

Current thinking in Nigeria and many low- and middle-income countries (LMICs) construes social health insurance schemes (SHIS) as one of the key mechanisms to achieving financial protection and universal health coverage (UHC) for its citizens [1]. High out-of-pocket expenditure (OOPE) remains a key factor working against achieving UHC in Nigeria, as OOPE is over 70% of total health expenditure, representing the highest in Africa [2].

In a bid to reduce high OOPE in Nigeria, a number of health reforms and health financing policies have been adopted. One of such reforms is the National Health Act signed into law in 2014 with a key aspect of a basic healthcare provision fund made up of not less than 1% of the federal consolidated revenue fund, which will partly (about 45% of the fund) be disbursed to all eligible States (in addition to the annual budget allocation to health) [3]. Rules and guidelines have been drafted for States to have access to these funds, which includes implementation of state SHIS. This follows the idea that the social health insurance (SHI) would provide financial protection, which would in turn reduce catastrophic OOPE whilst providing access to quality basic health services.

SHIS indeed have the potential to effectively help move a country in the direction of UHC by mobilising additional domestic resources for health through premiums/contributions, introducing essential organisational change needed for improved health system quality and efficiency, and providing better coverage through increased financial risk protection especially for the poor [4]. However, contributory health insurance schemes are not entirely new to the Nigerian context. Over the years, a national health insurance scheme (NHIS) and several community-based health insurance schemes have been implemented with mediocre results, such as extremely low coverage and failed/collapsed schemes due to a wide variety of issues, including low administrative capacity, small/fragmented risk pools and financial sustainability [5].

As a result of the implementation of health reforms in Nigeria, about 18 states have signed or are considering signing into law and adopting social health schemes. However, anecdotal evidence suggest that these states often lack the capacity to design an optimal and effective scheme. The issue of design is particularly important because literature suggests that variation within the key design features of SHI may explain failures, success or the speed at which UHC objectives are reached, thus informing both the readiness to implement or the likelihood of effectiveness of SHIS in each context [6].

In many contexts, the political and socioeconomic characteristics determine the options/choices of design of SHIS. For example, SHIS may be unrealistic in countries with stagnant economies and relatively large proportions of workers in the informal sector because collection of contributions can be extremely difficult, which means insufficient funds for the SHI and, consequently, financial unsustainability [7]. In addition, poorly designed and implemented SHIS have negative consequences, such as cost escalation and the tendency to divert resources from the poor to the rich [8]. Therefore, to ensure that the advantages of SHI outweigh its potential drawbacks, it is crucial to examine the suitability, readiness and potential problems before implementation of SHI in any given context. This foundation work may help states assess whether it is appropriate to proceed with SHIS or postpone SHIS until the necessary prerequisites for success are satisfied [9].

Given the likely urgency for states in Nigeria to implement SHIS, combined with the paucity of evidence within Nigerian states regarding SHIS and the potential for wastage of scarce resources due to collapse/risk of failure of such schemes, a simple checklist would be of immediate utility. This checklist can be readily applied by states to inform their preparation and design for the implementation of SHIS. In addition, the checklist would serve as guideline to inform states to maximise the chances of effectiveness of SHIS. This paper seeks to develop this checklist based on concepts as well as theoretical and empirical evidence.

## Methods

We conducted a review of the literature to explore the relevant concepts to develop a framework and checklist to identify the key factors or variables required to assess readiness/suitability of SHIS (Table 1). There were no strict inclusion and exclusion criteria. We included studies that described or evaluated SHIS across LMICs. Keywords were used to search electronic databases (such as PubMed, EMBASE, EconLit, and Google Scholar), grey literature, and websites of development partners such as WHO, the World Bank and USAID. Keywords used included contributory health insurance, social health insurance, pooling, revenue generation, strategic purchasing, risk pools, and provider payments. We also assessed bibliography of included studies to identify potential literature to inform our study.

**Table 1:** Social Health Insurance Checklist

Key design variables	Questions to be answered	Options			
Sources of finance	How would funds for the social health insurance (SHI) be generated/collected?	Premiums through social security contributions/payroll taxes/private contribution	Subsidies from taxes or other non-tax revenue	Others- donor funds/donations/co-payments	
	Is it pro-poor?	This option could potentially be pro-poor if contribution/participation is mandated and government pays subsidies for the poor	Whether this option is pro-poor is dependent on whether the taxes collected and used for SHI are progressive (pro-poor) or regressive (not pro-poor)	Donor funds have the potential to be directed towards the poor, but co-pays typically are not pro-poor unless there are mechanisms/waivers put specifically in place to protect the poor	
	Is collection feasible?	Collection for this option might not be feasible if the informal sector is large because it is difficult to collect taxes or social security contributions from small business or independent workers in the informal sector	Collection might not be feasible in developing contexts with narrow tax base, tax evasion and weak collection mechanisms	Yes	
	Will it be sufficient?	Funds generated from only this option are not likely to be sufficient if the informal sector is large because of the difficulty in collection of contributions; however, funds generated from a mix of all the available options might be sufficient but this depends on costed scenarios and benefit package offered	Funds generated from this option will likely not be sufficient if it is the major source of funds because of the difficulty of collection and competition from other sectors for government resources unless taxes/resources are earmarked specifically for SHI	No, if it is the major source of funds, especially in the long term	
Benefit package	What packages are offered	Comprehensive benefit package	Essential benefit package		
	Will the system have enough projected revenue to pay all its costs? (to be informed by a fiscal space analysis and a costing exercise)	Not likely/not encouraged, especially in poorer contexts where the SHI is often insufficiently funded because of weak mechanisms for collection	Yes, provided that benefit packages are properly costed based on projected financial resources, population health needs, infrastructure and utilisation rates		
Provider payment mechanisms	How are/will providers be organised and compensated?	Fee-for-service: charging a fee for each service offered to SHI members	Capitation: a fixed payment to providers per member enrolled to provide a defined package of benefits	Diagnosis-related groups: a form of per-case per-day hospital payment most commonly used to pay hospitals for inpatient treatment to treat a patient with a given diagnosis	Pay-for-performance/performance-based financing: is a value-based purchasing model that offers financial incentives to providers for meeting performance targets
	Are they efficient in cost containment to ensure high quality care is provided at the lowest possible cost?	No – High administrative costs driven by the difficulty in forecasting monthly or annual expenditure and the need for an elaborate information system with checks and balances to curb fraud There is perverse incentive for providers to maximise their income by increasing the number of services provided (supplier induced demand) and/or reducing the quality (and therefore the cost)	Very likely if designed properly – Lower administrative and transaction costs because payments are can be predicted accurately and made on regular schedule If enrolees have the opportunity to select their providers, there is an incentive for providers to attract enrolees to themselves by developing healthcare delivery innovations that improves quality of care	Not likely, unless the SHI organisation implements an elaborate monitoring system to control provider claims because there are perverse incentives for providers to maximise their income by keeping patients for longer days than required and submitting multiple claims for patients with comorbid conditions	Likely, as payment is upon achieving set of verified results; however, this might drive the administrative cost high as verification is needed prior to making payments to service providers The verification exercise is a means to curb an adverse incentive on the supply side of service
Contributing population and level of compulsion	Will membership be compulsory or voluntary?	Voluntary – Participation might be encouraged but no level of compulsion exists in participation	Mandatory – Individuals are compelled by law to enrol		
	Is it efficient for cross subsidisation?	No – Low compliance rates, which implies ‘low risk’ individuals may likely not join the scheme or only join when they fall ill; thus, leaving the risk pool composed mainly of high-risk individuals, placing financial strain on the SHI fund, leading to an unsustainable SHI	Yes – High compliance rate, which prevents exclusion of high-risk persons from membership, and protection against indirect risk selection		
	Feasibility of collection: are appropriate structures in place?	Yes – Because elaborate structures are not required to collect voluntary contributions	No – If the informal sector is large because it is difficult to collect taxes or social security contributions from small business or independent workers in the informal sector. Also, low buy-in from the formal sector might lead to resistance in mandatory contributions Mandatory contributions can only be successful if there is a clear connection between the new mandatory payments and increased benefits for those in the formal sector and strengthening information and collection systems for those in the informal sector		
Pooling of funds	Are funds combined in a single or multiple pool?	Single	Multiple (this may include presence of other pools/health insurance schemes, e.g. community-based health insurance schemes)		
	Is it efficient for risk equalisation/cross subsidisation?	Yes – Because cross-subsidisation/risk equalisation between high and low risk groups is easier in single/consolidated pools, which benefits from economies of scale	No – Because cross-subsidisation is difficult to achieve in fragmented or multiple risk pools Multiple risk pools could work in contexts where contributions are mandatory/compulsory to increase the size and mix of the pool		
Administration and management	Who will be responsible for oversight and monitoring the social health insurance system?	Private	Public		
	Are appropriate structures available to monitor and address issues relating to quality, utilisation, cost, efficiency and provider payments? This may require and organisational capacity assessment	Private management bodies are more likely to have more experience in administering and managing insurance schemes, with stronger skills and capacity listed: 1) The ability and information technology expertise to identify, register and enrol members from both formal and informal employees (determining which informal sector workers are to be exempted from contributions) 2) The ability and information technology expertise to routinely process and manage claims and payments to providers used by beneficiaries 3) Actuarial skills to budget, monitor and ensure that revenues are matched with likely expenditures 4) The expertise to set prices and manage cost inflation with health providers (negotiations with health providers, accreditation and provider payments) 5) Skills to investigate fraud, to ensure transparency and accountability of the SHI 6) Skills to define and refine the criteria for assessing the quality of health service delivery at individual health facilities	Not likely, especially in developing contexts where public bodies have weak administrative and organisational structure and exposed to political pressure, which could limit their capacity to make purely rational decisions in the best interest of the SHI Public management bodies can be efficient if qualified administrative personnel are hired and/or trained in the required competencies		

The checklist details critical considerations/questions to address and options for design. The developed checklist was then used to examine design features of SHIS in two states in Nigeria (Kaduna and Niger) between July and December 2018. The criteria for selection of the states included a constituted SHIS planning committee, public availability of draft bill, and commitment to evidence-based health financing strategies.

## RESULTS

### Conceptual framework

The conceptual framework consisted of four key design features – Sources of finance, Pooling (level of compulsion and risk pool), Strategic purchasing (benefit package, provider payment mechanism), and Administration and Management.

### Sources of finance

We found four important questions to be considered when thinking about the financing options for SHIS – How will the funds be generated, mobilised or collected? Is collection feasible? Is the source of finance pro poor? Will the funds generated be sufficient? We then identified three main sources of financing for SHIS – (1) premiums (through social security contributions, payroll taxes contributed typically by both employees and employers, and private contributions), (2) government subsidies (through general taxation, earmarked taxation or/and non-tax revenue), and (3) donor funds or other donations [10].

International experience suggests that it has proven extremely difficult to collect taxes or social security contributions from small business or independent workers in the informal sector. Thus, contributions from these sources alone might not be sufficient to fund SHIS, especially if the informal sector is large [11, 12]. Similarly, SHI schemes that are mainly funded through government subsidies obtained through taxes or other revenue tend to be underfunded and of poor quality [13, 14]. A possible explanation is that the health sector competes with other sectors for the same resources. This is particularly true in low- and middle-income contexts with a very narrow tax base [10, 15]. Evidence suggest that one of the ways to overcome underfunding in tax-funded SHIS is to earmark taxes (especially taxes generated progressively) to ensure protected funds for SHIS [16]. Direct taxes (e.g. income taxes) tend to be progressive, but indirect taxes (e.g. value added tax) are regressive, meaning that the poor pay more than their fair share. Indirect taxes have the potential to be progressive if they are imposed on goods purchased by the rich (luxury goods). If there is considerable potential to adjust the collection of indirect taxes to make it more progressive, then tax financing could be considered a viable option of SHIS financing that would not impose a greater burden on the poor [17].

Funds from loans and donations from international and multilateral organisations have also been shown to go a long way in financing SHIS, especially in the form of paying subsidies for the poor [18, 19]. However, there is strong evidence that suggests that these sources of funding are unsustainable in the long term, especially with donor funds declining steeply in the past few years, especially in developing contexts [20, 21].

It is also important to note that co-payments (or out-of-pocket payments) could be useful in some SHIS, provided that waivers for the poor are implemented. Co-payments can be either flat rate or a percentage of the fee. Apart from contributing to the SHIS funds, they help clients realise that they have the right to demand quality services because they are paying and they have the potential to help contain cost because they discipline clients to use appropriate levels of care in the health system and not to consult multiple providers for the same condition [17, 20].

Many countries do not rely on a sole source of revenue to fund their SHIS. Instead, they often tend to fund their SHIS with a mix of several sources outlined above, especially a combination of premiums from social security contributions or payroll taxes and subsidies from taxes or other non-tax revenue. Ensuring financial sustainability and raising sufficient funds for SHIS depend on (1) feasibility of collection of contributions from viable sources and (2) costing and planning for the benefit package that would be offered to the target/contributing population based on the projected fiscal resources and utilisation rates [22, 23].

In Rwanda for example, the *Mutuelle de Sante* health insurance scheme relies heavily on premium contribution at the community level as the primary source of financing. In 2006, there was a national policy review following backlash from critics whom highlighted the inequality associated with flat premium rates for its members. This eventually led to an introduction of premium subsidies for the poor and exemption for the poorest and an introduction of a ‘*ubudehe*’ process – a tiered fee system based on income categories for premium structures, which involved a poverty-mapping exercise that identified three categories of individuals, namely the poorest, middle-income earners and high-income earners [24].

### Pooling

Pooling is a function of the health system where resources for health are collected and further transferred to purchasing entities. The essence of pooling is to ensure the risk associated with financing of health interventions is shared amongst members of the pool as opposed to individuals to promote equity and efficiency. To a certain extent, collection of funds or premiums from enrolees depends on the level of compulsion of the scheme.

#### Level of compulsion of scheme

SHI schemes differ in the degree of obligation on individuals to participate in the scheme. Generally, participation could either be mandatory (where individuals are compelled by law to enrol) or voluntary (where participation might be encouraged but no level of compulsion exists in participation) [25, 26].

Voluntary membership often poses implications of adverse selection where ‘low-risk’ individuals may likely not join the scheme, as they may judge the premium to be excessive with respect to their health risks. In addition, some members might only enrol when they fall ill. Thus, leaving the risk pool composed mainly of high-risk individuals (with limited cross-subsidisation), placing financial strain on the SHIS fund [15, 26]. Since cross-subsidisation/risk equalisation is one of the guiding principles of SHIS, it is important that no population group is excluded, so that the risk pool is sustainable.

Experts have argued that the only way to improve participation in SHIS is to make it mandatory/compulsory, as evidence suggests weak enrolment compliance is a major barrier to increasing coverage rates among the near-poor and informal sector workers, and achieving UHC generally [15, 20, 26]. Breyer [25] further argued that the justification for compulsion lies in the understanding of SHI modelled after the Bismarck model, which is governed by the principle of equivalence between contributions and benefits (cross subsidisation). However, evidence suggests it is very difficult to collect meaningful levels of contributions from members from the informal sector. In addition, there is evidence of much slower implementation of SHI in contexts with rural population/informal sector [10, 15, 23]. Consequently, the ability to generate sufficient funds from mandatory SHIS may be difficult in contexts with a large informal sector and a high poverty rate. On the other hand, formal employees have a payroll system from which contributions can easily be deducted (through social security schemes or PAYE income tax system), compared to informal workers with variable and often undeclared income, especially in LMICs [15]. Another issue that could influence implementation of mandatory contribution is resistance of formal sector employees against ‘forcing’ them to buy-in to a scheme that might not necessarily present them with any advantages over the status quo [10, 15, 23].

#### Risk pool

The social insurance fund is generally viewed as the entity that combines funds from the population and assumes the function of financing a SHI system for the population. It is a concept that includes the idea of improving equity in access to services by mitigating the impact of out-of-pocket payments on the poor, the sick and the elderly. This can be achieved by spreading risk among members of a pool (pooling risks from the rich to the poor, from the healthy to the sick, and from the young to the old), which offers greater protection against high costs and thus improves financial accessibility and sustainability [10, 23].

The SHI fund can either be single or multiple funds. Single funds pool together the resources generated through various means. Advantages of this includes better risk equalisation between high- and low-risk groups (easier cross-subsidisation), minimal duplication of administrative duties, and reduced chance of provider fraud. Other advantages of single pools include benefits from economies of scale, which makes them potentially more efficient than fragmented systems [10]. On the other hand, multiple pools are not encouraged because they can contribute to the fragmentation of risk and inefficiencies. However, there may be logical reasons why single funds cannot exist. For example, decentralised governments with particularly autonomous provincial, district or state government pose challenges for pooling into a single fund [23]. In cases where there are subnational schemes with multiple risk pools, measures can be implemented to ensure risk equalisation and reduce fragmentation. Such measures include (1) mandatory contribution/enrolment to increase the size and mix of the pool and (2) pooling of funds from smaller community-based health insurance schemes and SHIS into one single pool at the state or district level [23]. This was implemented in the *Mutuelle de Sante* contributory health insurance scheme in Rwanda in 2012, when it was discovered that some districts experienced difficulties covering their expenses whilst others had surpluses, which led to the integration of funds into a single pool.

### Strategic purchasing

Purchasing in SHIS is an essential link between mobilised resources for health and effective delivery of quality services. This involves an active and evidence-based engagement to define the service mix and volume and selecting the provider mix in order to maximise national health priorities. The key features in strategic purchasing include defined service packages and a payment system that deliberately creates incentives for quality improvement [27].

#### Benefit package

A ‘benefit package’ refers to all the health services (and commodities) that would be offered to all SHIS enrolees/members (often with specific details of what is to be included and excluded). We found the critical consideration for optimal benefit packages is to include the sufficiency and affordability of the benefit package given financial resources, population health needs, infrastructure and utilisation rates. There are two broad categories of benefit packages typically offered, namely (1) an essential benefit package – covering basic primary healthcare services and occasionally a few related secondary health services, e.g. maternal and child healthcare, minor surgeries and minor illnesses; and (2) a comprehensive benefit package – covering a wide range of services across primary, secondary and emergency healthcare. This includes management of chronic conditions (e.g. diabetes) and major surgeries.

From global experience, essential benefit package tends to be the viable option in contexts with scarce resources and limited fiscal space. For this reason, many LMICs tend to start out with the essential package, before adding on more services, as the potential for the SHIS fund grows, whilst comprehensive packages are usually offered in richer contexts and/or countries where the SHIS is sufficiently funded either through earmarked taxes and a strong contribution collection system [22].

A major criterium for determining whether SHIS is feasible and financially sustainable depends on the range of services offered, proportion of total cost covered by the scheme and the population covered [10, 15]. Ultimately, it is important to cost whatever package is selected because funds available for SHIS are never unlimited. Costing the package will help in determining whether projected financial resources (based on potential contribution/collection rates) will be sufficient to fund the package, thus informing priority decisions regarding what can and cannot be covered, and for what reasons. This includes making trade-offs between cost-effective (value for money) options and population needs [17].

#### Provider payment mechanisms

Provider payment mechanisms address how providers are organised and compensated. A critical consideration for an optimal provider payment mechanism is to ensure high quality care is provided at the lowest possible cost. There are a number of provider payment mechanisms which include fee-for-service (FFS) payments, capitation payments, diagnosis-related groups and performance-based financing [17].

FFS payment involves the providers charging a fee for each service offered to enrolees. Providers submit bills for reimbursement and the SHI pays them. FFS payments tend to have high administrative costs and high potential for fraud. High administrative costs are driven by the difficulty in forecasting monthly or annual expenditure, the payment structure requires adequate skills and manpower to process payment, and an elaborate information system with checks and balances to ensure that providers are not submitting fraudulent bills [17, 20]. There is also a perverse incentive for providers to maximise their income under FFS payment system by increasing the number of services provided (supplier induced demand) and/or reducing the quality (and therefore the cost) of each service provided, which has implications for efficiency and innovations in service delivery [20, 23].

Capitation is a fixed payment to providers per member enrolled to provide a defined package of benefits. If designed and implemented properly, capitation payments are often desirable. There is a higher potential for cost containment under capitated payment arrangements compared to FFS for a number of reasons: (1) administrative costs are lower because capitation payments are fixed, payments can be made on a regular schedule because expenditures can be predicted accurately and (2) capitation payments implies fewer transactions compared to FFS, which limits transaction costs. Furthermore, when enrolees have the opportunity to select providers, there is competition among the providers, which creates better incentives for efficiency and incentives to develop healthcare delivery innovations that improves quality of care [20, 28].

Diagnosis-related groups is a form of per-case, per-day hospital payment most commonly used to pay hospitals for inpatient treatment. Providers examine the number of resources used (operating theatre, supplies, technology, drugs, medical staff and bed days) to treat a patient with a given diagnosis. The payments can be based on a flat rate per case or can differ depending on classes of diagnoses. Whilst diagnosis-related group provider payment is relatively easy to administrate, it has negative implications for cost control. For example, because a fixed fee per-case, per-day is received, providers might keep patients for longer days than required during recovery to earn more money. Another common example is provider submitting multiple claims for patients with comorbid conditions. However, the SHIS organisation can curb costs by implementing an elaborate monitoring system to control provider claims [10, 17].

Performance-based financing is an innovative, output-based approach where providers are paid based on agreed set of measurable performance targets. The goal is to improve on the use of health services by motivating providers to improve the quality of services provided [29]. However, lessons learned from countries suggest performance-based financing can be effective when public financial management systems and processes are flexible enough to ensure provider payments move to output payment [30].

A good example of utilising provider payment systems to contain cost is Ghana, where the NHIS initially started off with FFS, which encouraged wasteful practices such as overprescription of drugs to boost income of service providers, prolonged hospital days or unnecessary detention at hospital facilities, and encouraged frequent visits to the facilities. Following a reform of provider payment mechanism, capitation payments were introduced [31, 32].

Since SHI is often associated with high costs, it is important for the organisations running the system to contain costs, particularly by controlling adverse selection and moral hazard-induced behaviours that can be triggered by provider payment mechanisms. Purchasing and paying for services clearly requires an additional set of skills, particularly skills in contracting, setting expenditure caps and good monitoring of the system, among others, which need to be in place for successful implementation of SHIS [21].

### Administration and management of SHIS

Operating a SHIS entails several managerial and administrative tasks, which are critical in ensuring the financial sustainability of the scheme. This domain had two important considerations, namely (1) Who will be responsible for oversight and monitoring the social health insurance system? and (2) Is there adequate administrative capacity and management structures in place to effectively monitor and address issues relating to quality, utilisation, cost, efficiency and provider payments?

SHI and other health insurance schemes are often faced with an inherent uncertainty regarding their income and expenditure [17, 23]. For example, epidemics and other public health emergencies may temporarily increase utilisation rates, and hence expenditure; managing reserves to protect against this inherent uncertainty is an important measure for a health insurance to remain financially sustainable. Other important aspects of financial sustainability include cost containment (administrative and provider payments) and accountability, as evidence suggests administrative costs of over 8%, which have led to undesirable results [17].

Global experience suggests that, to ensure the functionality and success of SHIS, strong administrative capacity is required. This includes availability of personnel and structures for handling health insurance funds, overseeing its own operations, investigating fraud and complaints, negotiating, and sometimes contracting providers [15, 17, 26]. Specifically, the following capacities are required: (1) The ability and information technology expertise to identify, register and enrol members from both formal and informal sector (determining which informal sector workers are to be exempted from contributions); (2) The ability and information technology expertise to routinely process and manage claims and payments to providers used by beneficiaries; (3) Actuarial skills to budget, monitor and ensure that revenues are matched with likely expenditures; (4) The expertise to set prices and manage cost inflation with health providers (negotiations with health providers, accreditation and provider payments) [23]. Experts further suggest that a key component to strong administrative structure is a reformed Board of Trustees, which would include representatives from civil society with personnel responsible and reporting to the Board of Trustees on matters regarding (1) fraud and investigation, to ensure transparency and accountability of the SHI, (2) marketing, to develop and implement the communications strategy, and (3) benefits and quality, to define and refine standards of health services for enrolees and the criteria for assessing the quality of health service delivery at individual health facilities [33]. This is exemplified in the National Health Insurance Authority, which provides administrative support to the NHIS in Ghana through establishments of complaints committee, clinical audit units and/or a unit of the council is resident in every district office of the scheme to deal with conflicts and ensure quality services. Studies have shown that, since the establishment of a clinical audit in 2010 by the National Health Insurance Authority, it successfully identified weaknesses and challenges in quality and cost, which led to the recovery of over $11 million [34].

To ensure strong administrative capacity, it is essential that administrative personnel have appropriate educational qualifications and skills to plan and implement SHIS. It has been suggested that it is possible to utilise/leverage private bodies, as they tend to have better administrative experience with insurance schemes. However, these private bodies tend to drive administrative costs higher [35]. A counter argument by Somanathan et al. [10] proposes that, in most cases where public providers are used, there is a direct line of authority between the providers and the overseeing financing authority, which implies a simplicity of governance that provides the opportunity to organise the healthcare system more efficiently with lower transaction costs [10]. However, publicly managed SHI schemes are likely to be exposed to political pressure, which could limit their capacity to make purely rational decisions in the best interest of the SHIS [36]. Nevertheless, public sector management has the potential to be crucial to effective implementation if qualified administrative personnel are hired and/or trained. Whilst there are pros and cons for public or private administrative/managing bodies, in any case, it is essential to determine whether the capacity to run SHIS exists before establishing such schemes.

### The SHI checklist

Table 1 summarises and outlines the proposed SHI checklist detailing the design features described, critical questions to answer, and potential options to assess guide and inform the design of SHIS. For example, in the domain of sources of finance, which addresses the main question ‘how would funds for the SHIS be generated or collected’? The checklist presents the three main options, namely (1) premiums through social security contributions/payroll taxes/private contributions; (2) subsidies from taxes or other non-tax revenues, and (3) others – donor funds/donations/co-payments. We also present the critical considerations for the three options and evidence to support assessment – Is the option pro-poor? Is collection feasible? Will the funds generated from this option be sufficient?

It is important to note that, whilst we summarise evidence to help the checklist users assess and inform the design of their SHIS, addressing some critical considerations in a few design domains may require additional contextual formative research. For example, addressing the question of whether SHIS will have enough projected revenue to pay all its costs (benefit package domain) will likely require a costing study and a fiscal space analysis to assess the magnitude of the fund and what this fund could purchase.

### Results of application of the checklist on two SHIS in Nigeria

We report the results of the application of the checklist on planned SHIS in two states in Nigeria (Kaduna and Niger), which is summarised in Table 2. Data used to inform the checklist include draft health insurance bills and consultations with SHIS planning committee and planners, which included, but were not limited to, officials from the State Ministry of Health, State Primary Health Care Development Agency and representatives from the NHIS.

**Table 2:** Results of application of the checklist to social health insurance scheme (SHIS) design in Kaduna and Niger States

Key design variables	Question to be answered	Kaduna	Niger
Sources of finance	How would funds for the SHIS be generated/Collected	• Initial take-off grant • Equity contribution of 1% consolidated revenue fund • Contribution from employers, employees in public and private sector • Contributions from informal sector • Contributions from students in tertiary institutions • Funds from the national health insurance scheme (NHIS) for pregnant women, children under 5 • Donations • Appropriations earmarked for implementation of scheme • Fines and commissions charged by agency • Dividends and interests on investments	• Initial take-off grant • Equity fund of 1% consolidated revenue fund • Formal sector contribution of public and private employers and employees • Informal sector contribution • Funds from NHIS for pregnant women and children under 5 • Donations or grants • Fines and commissions charged by the agency • Appropriations earmarked for implementation of the scheme • Dividends and interests on investments and stocks
	Is it pro-poor?	The scheme appears to be pro-poor as there is an equity fund established for the vulnerable groups	The scheme appears to be pro-poor as there is an equity fund established for the vulnerable groups
	Is collection feasible?	Most likely; although, government funds are dependent on availability of funds/budget release. There is, however, no mechanism to collect contributions from informal sector	Most likely for most part; although, the informal sector will be more challenging
	Will it be sufficient?	Not likely; a fiscal space for health is ongoing. Compliance rate will determine how sufficient the funds will be	Several factors will determine how sufficient it will be; compliance rate and budget release
Benefit package	What packages are offered	Essential services	A mix of essential and/or comprehensive packages will be offered depending on the health plan.
	Will the system have enough projected revenue to pay all its cost?	Most likely; provided the benefit packages are well costed based on population needs and utilisation rates	Not likely; a major source of fund needs to be established with adequate capacity to collect contributions. Adequate costing done for the different health plan package of service
Provider payment mechanism	How will providers be organised and compensated?	Discussions are ongoing Capitation/performance-based financing (PBF) will be adequate since it is one basic plan for all	Discussions are ongoing; however, a mix of capitation and PBF can be proposed for outpatient and inpatient services, respectively
	Are they efficient in cost containment to ensure high quality care is provided at the lowest possible cost?	If designed properly, yes. The state is providing a basic health plan to all members of the scheme. Either capitation/PBF can curb cost and provides an incentive for provider to offer quality service. Although capitation runs a risk of providers neglecting clients too.	Most likely especially if designed properly; although PBF might be associated with high administrative cost due to verification exercise but it can be merged to the activities of the scheme
Contributing population and level of compulsion	Will membership be compulsory or voluntary?	Mandatory for all residents	Mandatory for all residents
	Is it efficient for cross subsidisation?	Not likely; although if the compliance rate is high there is a chance of efficient cross subsidisation. In addition, if the subsidies for the vulnerable are pooled to the fund	Most likely only if the compliance rate is high
	Feasibility of collection: are appropriate structures in place?	The scheme has no appropriate structures in place to collect contributions from informal sector and the formal sector might resist	The scheme has no appropriate structures in place to collect contributions from informal sector and the formal sector might resist
Pooling of funds	Are funds combined in a single or multiple pool?	Single centralised pool	Single centralised pool
	Is it efficient for risk equalisation/cross subsidisation?	Yes – Provided compliance rate is high; it means both low- and high-risk groups are within the pool	Yes – Provided compliance rate is high; it means both low- and high-risk groups are within the pool
Administration and management	Who will be responsible for oversight and monitoring the social health insurance system? (Administrative autonomy)	Executive secretary of the agency will provide oversight An actuary will be responsible for benefit packages	Executive secretary of the agency will provide oversight An actuary will be responsible for benefit packages
	Are appropriate structures available to monitor and address issues relating to quality, utilisation, cost, efficient and provider payments?	Uncertain; although the actuary is an independent consultant most likely from the private sector Tasked with the responsibility of reviewing benefit packages, utilisations and contributions	Uncertain; although the actuary is an independent consultant most likely from the private sector Tasked with the responsibility of reviewing benefit packages, utilisations and contributions

**Sources of Finance** In both states, plans for revenue generation/collection appears to be form multiple options. It was reported that, to ensure all infrastructure needed are put in place, an initial take-off grant will be provided by the government and subsidies will be offered to vulnerable groups, including pregnant women and children under 5 years of age, through equity contributions. In addition, the organised formal sector employees and employers are expected to contribute a percentage to cover the full cost of their premiums. Given the ease through automation in the formal sector, collection of the premiums appears feasible. The states also indicate they intend to collect contributions from the informal sector, but there are no clear stated mechanisms to ensure this collection.

**What benefit packages are offered?** Niger state proposes offering a prescribed benefit package that differs based on enrolee’s willingness to pay, which consists of a variety of packages providing extra healthcare services in direct proportion to the contribution made. However, Kaduna is considering opting for a single plan offering essential/basic services to all members.

**What provider payment mechanism is in place?** At the time of the study, in both states, there were no specified provider payment mechanisms in place and discussions are ongoing regarding the best approach.

**What is the contributing population and level of compulsion?** Both states propose mandatory participation for all residents of the state. At the start of the scheme, the contributing population will be limited to the formal sector workers in public and private institutions and students in tertiary institution as seen in Niger state. As at 2018, only about 6% of Kaduna’s working population were formally employed, whilst 36% were informally employed. Similarly, in Niger, only 4% of the population were formally employed, whilst 41% were informally employed in 2018 [37]. Both states plan on leveraging community outreaches and awareness programmes to enlighten the populace on the scheme to ease acceptance.

**Pooling of funds: will the SHIS funds be pooled into single or multiple pools?** Both states intend to operate a consolidated pool. The single pool encourages cross subsidisation. However, there are no mechanisms in place for the states to integrate existing community-based health insurance schemes in Niger and Kaduna states.

**Administration and management** The two states assessed have proposed to have a standalone public agency to manage and implement the SHIS. Based on the draft SHIS bills in the two states, an executive secretary will be appointed, and their sole responsibility is providing oversight and management to the scheme. There will also be a Board of Directors, but the extent of their influence and details of administration are unclear.

## Discussion

The paper aimed to develop a simple evidence-based checklist to aid Nigerian states in designing successful contributory health insurance schemes. The developed checklist can also be applied across many different contexts, especially LMICs, to serve as a guide to inform policy-makers, researchers and others about the readiness to implement SHIS as it relates to design features. The checklist builds on theoretical and empirical evidence on the key insurance design components and their relevance in the wider health system. However, this checklist is not without its limitations. First, it does not provide a comprehensive guide on how to solve inefficiencies relating to design. In addition, the checklist is only limited to design variables even though there are other critical factors, such as legislature and political economy, that could enable success and sustainability of a SHIS.

Despite the limitations of the checklist, it presents an important first step towards the use of evidence to inform SHIS design. The checklist consists of six domains that focus on the source of funds, benefit packages, provider payment mechanisms, contributing population and level of compulsion, pooling of funds, and administration and management of the insurance schemes.

Regarding the sources of funds, the SHIS in both states appeared to be pro-poor, in that equity funds are planned to be used to subsidise premiums for vulnerable groups. In addition, there is proposed mandatory participation for all residents of the state. Even though the method of mandating participation is yet to be established, evidence suggest that compelling the large informal sector to contribute premiums may be difficult. Given that mandatory contributions present preferable options for raising sufficient funds and risk equalisation, key measures need to be in place for effective implementation to improve compliance and ensure efficient collection. These include education and advocacy to increase buy-in of the formal sector (SHI schemes might only be politically feasible if there is a clear connection between the new mandatory payments and increased benefits for those who pay), strengthening information systems (improving information exchange with business-registering authorities for registration and with tax revenue authorities for contribution collection), and strengthening governance and organisation of collection [10, 26].

The benefit packages being proposed in both states were essential benefit packages, which seems reasonable given the fiscal constraints in these states [38, 39]. However, it is important to cost whatever package is selected because funds available for SHI are never unlimited. Costing the package will help in determining whether projected financial resources (based on potential contribution/collection rates) will be sufficient to fund the package, thus informing priority decisions regarding what can and cannot be covered, and for what reasons. This includes making trade-offs between cost-effective (value for money) options and population needs [17]. In addition, defining the benefit package is not a one-off but a continuous process to review health priorities and population needs that align with health systems objectives and target interventions to services that offer the highest value.

At the time of the study, there were no specified provider payment mechanisms in place and discussions are ongoing regarding the best approach in the assessed states. Options presented in our checklist suggest that provider payment systems should prevent waste and unnecessary service provision. In addition, as some providers are already being paid by the NHIS in these states, the States can leverage on lessons learnt and institutional memory. As Nigerian states begin to design and implement SHIS, it is important that they carefully review and select provider payment options that will optimise cost containment and improve quality of care. This may require an additional set of skills, particularly skills in contracting, setting expenditure caps and good monitoring of the system, among others, which need to be in place for successful implementation of SHIS [21].

Regarding the administration and management of the scheme, both states assessed have proposed to have a standalone public agency to manage and implement SHIS, but the mode of operation and extent of influence are unclear. Whilst evidence suggest that there are pros and cons for public or private administrative/managing bodies, it is essential, in any case, to determine whether the capacity to run SHIS exists before establishing such schemes. This includes ensuring qualified administrative personnel are hired and/or trained, with the ability to navigate and address issues such as (1) processing and managing claims and payments to providers used by beneficiaries, (2) setting prices and managing cost inflation with health providers, and (3) fraud and investigation to ensure transparency and accountability.

## Conclusion

Given that the development of SHIS in the Nigerian states is still in its early stages, applying the SHIS design checklist can serve as a first step to design a feasible and sustainable insurance scheme. Whilst this checklist may serve as an important first step towards designing a sustainable SHIS, the checklist may be improved and further refined and informed by future research, which may include evaluation of the checklist and expert consultations. Prior to the SHIS, Nigeria had operated different forms of contributory health insurance schemes and inefficiencies were prominent, leading to poor coverage despite all efforts to expand the scheme. The introduction of SHIS, if properly designed and implemented, can be a significant reform with the potential to improve the accessibility, equity and efficiency of healthcare in Nigeria.

## Data Availability

Not applicable.

## Abbreviations

CBHI: community-based health insurance
FFS: fee-for-service
LMIC: low- and middle-income country
NHIS: National Health Insurance Scheme
OOPE: out-of-pocket expenditure
SHI: Social Health Insurance
SHIS: Social Health Insurance Scheme
UHC: universal health coverage
